# Integration of multiomics with precision nutrition for gestational diabetes: Study protocol for the Westlake Precision Birth Cohort

**DOI:** 10.1002/imt2.96

**Published:** 2023-03-15

**Authors:** Xinxiu Liang, Zelei Miao, Sha Lu, Meng Ye, Jiali Wang, Hui Zhao, Congmei Xiao, Menglei Shuai, Wanglong Gou, Yuhui Liang, Fengzhe Xu, Mei‐Qi Shi, Ying‐Ying Wu, Xu‐Hong Wang, Feng‐Cheng Cai, Meng‐Yan Xu, Yuanqing Fu, Wen‐Sheng Hu, Ju‐Sheng Zheng

**Affiliations:** ^1^ Westlake Intelligent Biomarker Discovery Lab Westlake Laboratory of Life Sciences and Biomedicine Hangzhou China; ^2^ School of Life Sciences Westlake University Hangzhou China; ^3^ Institute of Basic Medical Sciences Westlake Institute for Advanced Study Hangzhou China; ^4^ Department of Obstetrics and Gynecology Hangzhou Women's Hospital (Hangzhou Maternity and Child Health Care Hospital) Hangzhou China; ^5^ Department of Obstetrics and Gynecology The Affiliated Hangzhou Women's Hospital of Hangzhou Normal University Hangzhou China; ^6^ Department of Nutrition Hangzhou Women's Hospital (Hangzhou Maternity and Child Health Care Hospital) Hangzhou China; ^7^ Department of Nursing Hangzhou Women's Hospital (Hangzhou Maternity and Child Health Care Hospital) Hangzhou China

## Abstract

We established a prospective birth cohort, the Westlake Precision Birth Cohort (WeBirth), based on 2000 pregnant women with gestational diabetes mellitus (GDM) in the second trimester and their offspring. The WeBirth provides a new framework for prospective birth cohort study with sophisticated integration of precision nutrition, wearable devices, and multiomics data collection among patients with GDM.

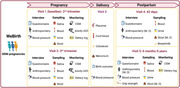

## INTRODUCTION

Gestational diabetes mellitus (GDM) is a common pregnancy complication, and its prevalence has been increasing worldwide [1]. Specifically, the prevalence of GDM has exceeded 20% in Asian and African countries [2], posing a huge medical and economic burden. Women with GDM are at an increased risk of developing short‐ and long‐term complications, such as hypertensive disorders during pregnancy [3], perineal trauma [4], and type 2 diabetes (T2D) [5]. Meanwhile, maternal glucose intolerance may promote fetal glucose intake [6], leading to higher risk of large‐for‐gestational‐age (LGA) birth or macrosomia [7], neonatal hypoglycemia [8], and metabolic syndrome during childhood and later life [9].

Lifestyle interventions (including nutrition and physical activity) represent the first‐line therapy for GDM [10, 11, 12]. Pregnancy nutritional status has a substantial influence on the health status of the women themselves and their offspring [13, 14]. However, it is also well known that different individuals may respond differently to the same diet or nutritional exposures. For example, postprandial response of glucose or other metabolic traits to the same food has been found to be varied among nonpregnant individuals [15, 16]. So far, personalized glycemic responses of pregnant women with GDM to diet/nutritional interventions and their relationship with birth outcomes have not been investigated. Here, we summarized more than 40 birth cohorts worldwide, including sample sizes, strengths, and limitations (Table S1). As we can see, most of these birth cohorts included general pregnancies with large sample sizes. However, multiomics measurements were lacking.

Understanding personalized glycemic responses of pregnant women to different dietary challenges can help establish precision nutrition recommendations for pregnant women with GDM, which can further improve the health of these women and their offspring [17, 18]. The emerging technology of continuous glucose monitoring (CGM) makes it convenient for us to monitor glucose fluctuations, postprandial glycemic responses, hyperglycemic and hypoglycemic status, and glycemic variability [19]. Therefore, we have established a birth cohort (Westlake Precision Birth Cohort [WeBirth]) based on pregnant women with GDM, integrating the utilization of wearable devices including CGM and objective measurements of physical activity, standardized testing meals, daily dietary records, and collection of multi‐omics data. The purpose of the standardized meal tests is to examine the personalized response to the diet. Our main objective is to explore optimal nutrition recommendations for patients with GDM from the perspective of precision nutrition and the association between personalized blood glucose response and birth outcomes.

## COHORT DESIGN

**Figure 1:**
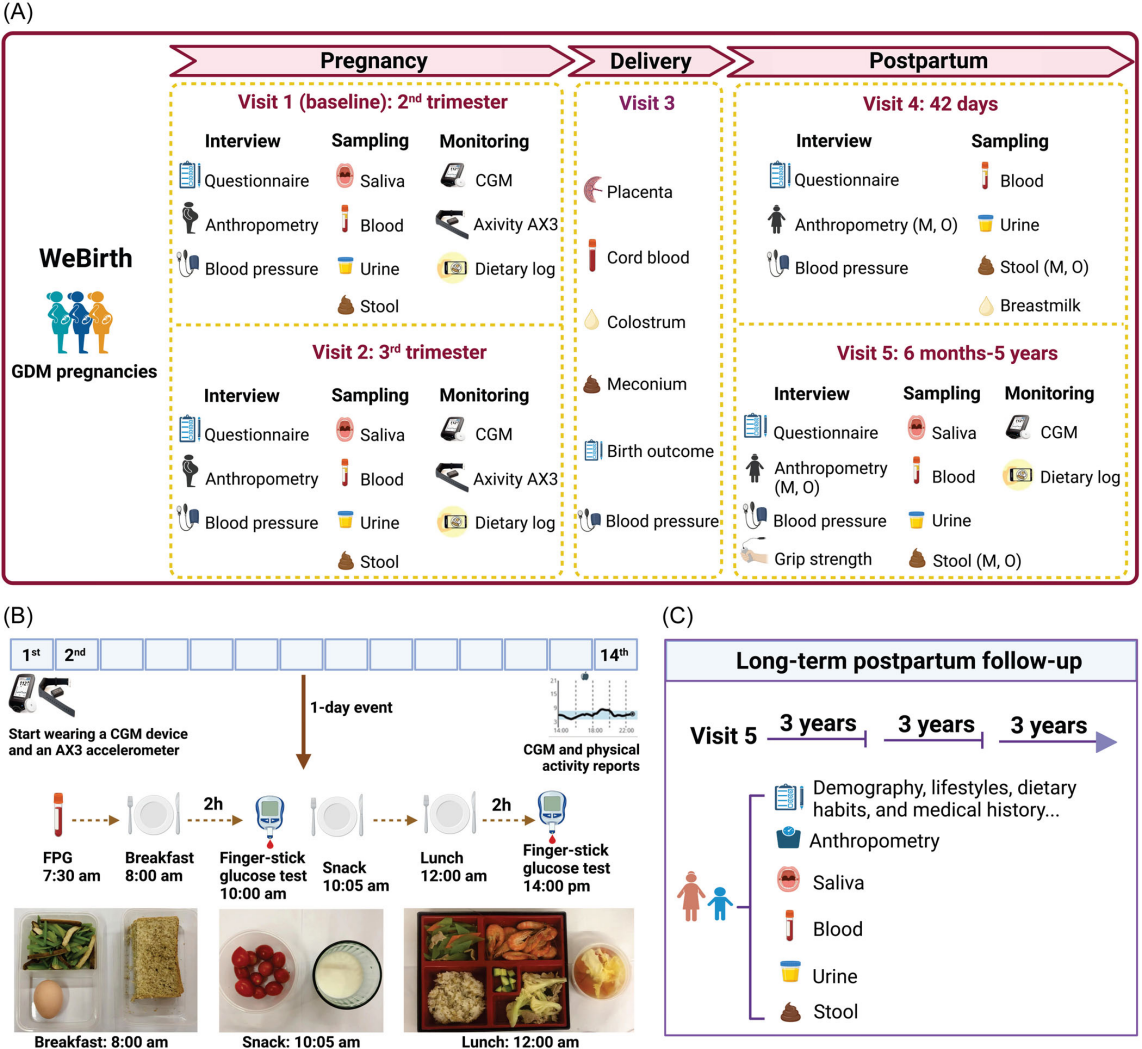
Study design of WeBirth. (A) Overview of the study design. (B) Standardized testing meal during the 1‐day visit of the participants to the clinical center. (C) Longitudinal follow‐up. Both the mothers and the children will be followed every 3 years after visit 5. The image was created with BioRender.com. Axivity AX3, Axivity AX3 accelerometer; CGM, continuous glucose monitoring; FPG, fasting plasma glucose; GDM, gestational diabetes mellitus; M, mother; O, offspring; WeBirth, Westlake Precision Birth Cohort.

WeBirth is designed as a prospective cohort study among pregnant women with GDM and their offspring in Hangzhou, China. Figure 1A shows the overview of the study design. In WeBirth, we incorporate three unique components, (1) objective measurement of physical activity during pregnancy using the Axivity AX3 accelerometer (Axivity Ltd.); (2) measurement of dietary intake using both the Food Frequency Questionnaire (FFQ) and a dietary log/record; and (3) measurement of blood glucose levels over 14 days using CGM (Freestyle Libre Pro), with standardized testing meals during the 14‐day period.

The primary aims of WeBirth are to investigate:

1. The continuous blood glucose response to daily dietary intake and physical activity, and to facilitate the development of personalized nutritional/lifestyle recommendations among patients with GDM.
2. The association of dietary intake and physical activity with continuous glucose fluctuations during pregnancy on adverse birth outcomes, such as preterm birth, low birth weight (or small‐for‐gestational‐age birth), and macrosomia (or LGA birth).

The secondary aims of WeBirth are to investigate:

1. Potential associations of CGM during pregnancy with the body composition of the adult participants after delivery and weight changes of the offspring during early childhood.
2. Risk factors during pregnancy (e.g., continuous glucose characteristics, dietary habits, and oral and gut microbiome) for developing T2D after delivery among women with GDM.

## BASELINE RECRUITMENT

Our planned sample size (*n* = 2000) was based on the literature of precision nutrition studies for multi‐omics analyes [15, 16]. WeBirth includes pregnant women attending prenatal care at the Hangzhou Women's Hospital (Hangzhou Maternity and Child Health Care Hospital), China. The recruitment has been initiated since August 2019. Inclusion and exclusion criteria are described below:

*Inclusion criteria*

1. Pregnant women aged 18 years or older.
2. Pregnant women diagnosed with GDM, with a gestational age mainly ranging from 24 to 28 weeks.
3. Pregnant women intending to deliver at Hangzhou Women's Hospital.
4. Pregnant women intending to remain in Hangzhou with their child for ≥4 years.

*Exclusion criteria*

1. Pregnant women with cancer or other serious medical disorders.

During routine prenatal care, a 2‐h 75‐g Oral Glucose Tolerance Test is performed on women at 24–28 weeks of pregnancy. We use the International Association of Diabetes and Pregnancy Study Groups criteria for GDM diagnosis (fasting plasma glucose [FPG] ≥ 5.1 mmol/L, and/or 1‐h plasma glucose ≥10.0 mmol/L, and/or 2‐h plasma glucose ≥8.5 mmol/L) [20].

Our participants are recommended by a nutritionist. After being diagnosed with GDM, pregnant women come to the department of nutrition for dietary counseling on the basis of the recommendation of the obstetrician. The nutritionist conducts an initial screening to exclude those unwilling to wear a CGM device or with extremely poor compliance, and then recommends the remaining patients to our room for the interview. A trained staff further estimates whether their conditions meet the inclusion criteria and their willingness to participate. The median interval between GDM diagnosis and the recruitment into the present study is 0 day (quartile [Q]1 = 0; Q3 = 6). During the baseline visit (visit 1), the staff administer the in‐person interview with the participants using different questionnaires to collect information on demography, lifestyle, dietary habits, physical activity, occupational history, and history of medications and family disease. Bone mineral density and body composition are assessed using an ultrasound bone densitometer (Sunlight MiniOmni, BeaMed Co.) and the InBody 720 (Biospace Co., Ltd.), respectively. Each individual is asked to wear a CGM device and an AX3 accelerometer for 14‐day blood glucose and physical activity monitoring, respectively. We randomly select some participants to wear CGM devices on both two arms at the same time to verify the stability of the readings. Participants record their dietary intake and nutritional supplements (with an accompanying picture) on an online APP, while wearing the CGM device and the accelerometer. The APP has been developed to support the present study as an electronic dietary log. Meanwhile, the participants report their food intake of the previous day using the 24‐h Dietary Recall (24 HR) at recruitment. Biological samples of saliva, blood, urine, and stool are collected.

## STANDARDIZED TESTING MEALS

**Figure 2:**
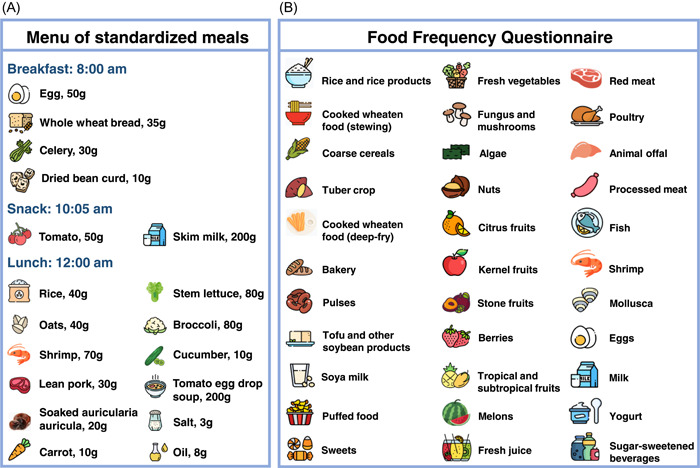
Food items of the standardized meals (A) and the Food Frequency Questionnaire (FFQ) (B).

As part of routine health counseling for the pregnant women with hyperglycemia at the Hangzhou Women's Hospital, all these participants are invited to attend a 1‐day event/lesson about diet and lifestyle modifications to manage their glycemic status during pregnancy (Figure 1B). The standardized testing meals were developed by the nutritionist according to the Dietary Guide for Patients with Gestational Diabetes Mellitus from the National Health Commission of the People's Republic of China (WS/T 601—2018). During the 1‐day event, the standardized meals are prepared in the staff cafeteria of the hospital using standard ingredients: egg, whole wheat flour, celery, and dried bean curd for breakfast; tomato and skim milk as snacks; and rice, oats, shrimp, lettuce, lean pork, carrot, soaked auricularia auricula, broccoli, cucumber, and tomato egg drop soup for lunch (Figure 2A). On the morning of the 1‐day event, fasting blood samples (≥8‐h fasting) are obtained from the participants to measure the levels of FPG, HbA1c, and vitamin D. Then all the participants are located in an assigned room within the hospital, and have standardized breakfast, snacks, and lunch there. Finger‐stick glucose tests are conducted twice after fasting for a 2‐h period after breakfast and lunch. Trained nurses/physicians provide pregnancy‐related health knowledge to participants through talks between the meals.

## FOLLOW‐UP VISITS

We follow the participants during the third trimester, during delivery, and after delivery (Figure 1A). During the third trimester (visit 2), a follow‐up interview takes place when the pregnant women visit the hospital for prenatal care. We mainly collect data about sleep quality and psychological health using questionnaires at this interview. Simultaneously, repeated measurements of blood pressure, weight, CGM, physical activity, and 24 HR are performed. Biological samples are collected similar to those at baseline. During delivery (visit 3), information on birth outcomes (e.g., gestational age at delivery, birth weight, height, and perinatal medical information) is obtained from the medical records of the prenatal care and delivery clinic. Biological samples of placenta, cord blood, meconium, and colostrum are collected during visit 3.

The postpartum follow‐up 42 days after delivery (visit 4) is conducted in the hospital when the participants visit the hospital for postnatal care, and mainly, information on feeding practices and growth and development of the infants is collected. Due to the covid pandemic and occasional local virus outbreak and government control, our planned postpartum follow‐up at the home of the participants has been considerably disrupted. Therefore, the originally planned follow‐up at 6–18 months will have to be modified to follow‐up at 6 months to 5 years accordingly for visit 5. The follow‐up of visit 5 will be divided into a home visit and a hospital visit. During the home visit, the mothers will fill out a questionnaire aimed at collecting information on dietary habits, lifestyle, and medical history of themselves and their offspring. These mothers will be asked to wear a CGM device again for 14 days and record their daily dietary intake using the APP at the same time. Physical examination and biological sample collection of these women participants will take place at the physical examination center of the Hangzhou Women's Hospital. Anthropometric parameters of the offspring will be measured at the Children's Hospital of Zhejiang University School of Medicine. These children will also undergo different developmental screenings according to their ages, such as hearing screening.

Although we will try our best to follow up these participants, loss to follow‐up will still be a major issue in a cohort study. Therefore, we will try to encourage the return of these participants to the study center using different strategies, such as special health consulting lessons about child rearing and free health checkups for both mothers and children.

As pregnant women with GDM are at high risk of developing T2D after delivery and the offspring are likely to suffer from cardiometabolic diseases during growth [21], we will continue to follow these participants and their offspring longitudinally (Figure 1C). The long‐term follow‐up will take place every 3 years. Information and biological samples similar to those at visit 5 will be collected during long‐term postpartum follow‐ups. The long‐term follow‐up appointment will also include two sections: a home visit and a hospital visit. During the home visit, we will collect information on the dietary habits, lifestyle, and medical history of both the mothers and the offspring using questionnaires. Additionally, physical examination of both the mothers and the children will be conducted at the hospital.

## QUESTIONNAIRE‐BASED SURVEY

**Figure 3:**
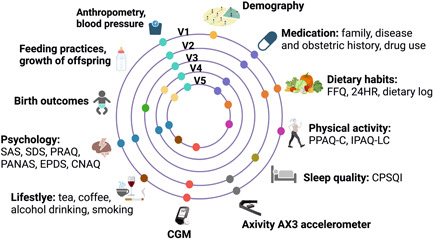
Data collection during interviews. V1‐V5 represent visit 1 (baseline, second trimester), visit 2 (third trimester), visit 3 (delivery), visit 4 (42 days after delivery), and visit 5 (6 months–5 years after delivery), respectively. Different colored points on the circle trajectories represent the corresponding data collection during each visit. The image was created with BioRender.com. 24 HR, 24‐h Dietary Recall; CGM, continuous glucose monitoring; CNAQ, Council on Nutrition Appetite Questionnaire; CPSQI, Chinese version of Pittsburgh Sleep Quality Index; EPDS, Edinburgh Postnatal Depression Scale; FFQ, Food Frequency Questionnaire; IPAQ‐LC, International Physical Activity Questionnaires‐long, Chinese; PANAS, Positive and Negative Affect Scale; PPAQ‐C; Pregnancy Physical Activity Questionnaire, Chinese; PRAQ, Pregnancy‐Related Anxiety Questionnaire; SAS, Self‐rating Anxious Scale; SDS, Self‐rating Depression Scale.

The questionnaires have been developed to collect data about demographics, lifestyle, dietary habits, physical activity, occupational history, medical and family disease history, feeding practices, and growth and development of the infants (Figure 3; detailed information is provided in Table 1). Participants complete the questionnaires under the guidance of the staff during face‐to‐face appointments, except for the questionnaire used to collect information on medical and family disease history at baseline. We have developed an FFQ (Figure 2B) containing 33 items of food/food groups to collect information on dietary habits of our participants, considering the regional characteristics (the Yangtze Delta of China) of dietary intake and the existing questionnaires from Chinese cohorts, including the Guangzhou Nutrition and Health Study [22]. We apply the Chinese version of the Pregnancy Physical Activity Questionnaire (PPAQ‐C) [23] and the International Physical Activity Questionnaires, long form (IPAQ‐LC) [24] to obtain information on physical activity of participants during pregnancy and after delivery, respectively. The Chinese version of Pittsburgh Sleep Quality Index [25] is used to assess multiple dimensions of sleep quality of participants. Moreover, we utilize the Chinese version of the Self‐rating Anxious Scale [26], the Self‐rating Depression Scale [27], the Pregnancy‐Related Anxiety Questionnaire [28], the Positive and Negative Affect Scale [29], the Edinburgh Postnatal Depression Scale [30], and the Council on Nutrition Appetite Questionnaire [31] for the assessments of psychosocial characteristics, including anxiety, depression, mood (or emotion), and appetite, at different stages of the appointments. All the questionnaires are constructed through on an online platform (“Survey Star,” Changsha Ran Xing Science, and Technology) with the automatic warning of missing data or extremely outrageous value for each variable.

**Table 1:** Information collected during interviews.

	Pregnancy		Visit 3: delivery	Postpartum	
	Visit 1 (baseline): second trimester	Visit 2: third trimester		Visit 4: 42 days	Visit 5: 6 months–5 years
*Demographic characteristics*	✓				
*Anthropometric characteristics*					
Basic anthropometric characteristics	✓	✓	✓	✓	✓
Bone mineral density	✓				
Body composition	✓				
*Blood pressure*	✓	✓	✓	✓	✓
*Wearable devices*					
Continuous glucose monitoring	✓	✓			✓
Axivity AX3 accelerometer	✓	✓			
*Medication*					
Family and disease history	✓				✓
Obstetric history	✓				
Drug use	✓	✓		✓	✓
*Health behavior*					
Food Frequency Questionnaire	✓				✓
24‐h Dietary Recall	✓	✓			✓
Dietary log	✓	✓			✓
Pregnancy Physical Activity Questionnaire, Chinese	✓				
International Physical Activity Questionnaires‐long, Chinese					✓
Chinese version of Pittsburgh Sleep Quality Index		✓			
Tea, coffee, alcohol drinking and smoking	✓				✓
*Psychological characteristics*					
Self‐rating Anxious Scale	✓				✓
Self‐rating Depression Scale	✓				
Pregnancy‐Related Anxiety Questionnaire		✓			
Positive and Negative Affect Scale		✓			
Edinburgh Postnatal Depression Scale				✓	
Council on Nutrition Appetite Questionnaire	✓				
*Offspring health*					
Birth outcomes			✓		
Feeding practices				✓	✓
Growth and development of offspring				✓	✓

## BIOLOGICAL SAMPLING PROCEDURES

Figure 1 presents an overview of biological sample collection across different time points of WeBirth. Saliva samples are collected at the hospital (visits 1 and 2) or at home (visit 5) using tubes with a DNA stabilizer and transferred to a freezer (−40°C) within 10 h after production. Before collecting saliva samples, participants need to wait at least 30 min after eating, drinking, smoking, or chewing, and drink some water to clean the mouth.

Fecal samples from pregnant women are collected at the hospital during pregnancy, aliquoted, and stored at −40°C within 1 h after production. We preserve three types of fecal aliquots, including raw feces, feces mixed with an RNA stabilizer, and feces mixed with 50% glycerin. Meconium and colostrum are collected within 3 days after delivery, kept at 4°C temporarily, and transferred to a −40°C freezer within 15 h of collection. For the postpartum visits, fecal samples from mothers or their offspring are collected into tubes with a DNA stabilizer at home and transferred (room temperature) to the laboratory for aliquot and storage at −40°C within 3 days of collection. Specimens of midstream urine and breastmilk are collected at the hospital, immediately placed at 4°C, aliquoted, and stored at −40°C within 3 h of collection.

All serum samples and most whole‐blood samples are collected after at least 8 h of fasting (whole‐blood samples of the third trimester are sampled randomly). All blood samples are kept at 4°C temporarily after collection. Then, plasma, white cell, red cell, and serum samples are aliquoted and stored in a −40°C freezer.

Cord blood samples are collected by nurses using lavender stopper BD Vacutainer K2EDTA tubes in the delivery room. Two fragments (1 cm^3^) of fetal‐surface placenta are collected within 1 cm from the umbilical cord. Samples of cord blood and placenta are kept at 4°C immediately and stored at −40°C within 24 h after collection. Placenta samples are mixed with a DNA stabilizer before being transferred to a −40°C freezer.

All the biological samples mentioned above are frozen temporarily at −40°C in the hospital. Within 1 month of collection, all the biological samples are shipped on dry ice to Westlake University to be stored at −80°C.

## METHODOLOGIES OF BIOLOGICAL SAMPLE PROCESSING

For blood samples, measurements of common clinical biomarkers, such as cell types and counts, blood glucose, blood lipids, and markers reflecting liver, kidney, gallbladder, and thyroid functionalities, are assessed using automatic equipment in the laboratory of the hospital. Additionally, blood samples of some of the participants have been used for genomics and proteomics according to the existing protocols. For genomics, DNA is extracted from clotted blood using the TIANamp Blood DNA Kit (DP348, TianGen Biotech Co., Ltd.). DNA concentrations are determined using the Qubit quantification system (Thermo Fisher Scientific). Extracted DNA is stored at −80°C until further assessment. We apply Illumina ASA‐750K arrays containing 700 thousand sites for genotyping and PLINK1.9 for quality control and relatedness filters. Serum proteomics is performed using the SWATH‐MS method [32]. Following the protocol, peptides are digested from proteins, cleaned, and then analyzed using SWATH‐MS over a 20 min linear LC gradient on a TripleTOF 5600 system (SCIEX) coupled to an Eksigent NanoLC 400 58System (Eksigent).

Saliva and fecal samples are mainly intended for microbiome measurements, including 16S rRNA gene sequencing, metagenomics, and internal transcribed spacer 2 (ITS2) sequences. The protocols of microbiome analysis have been established. The microbiome DNA is extracted using the QIAamp DNA Stool Mini Kit (Qiagen) according to the manufacturer's instructions. DNA quality is determined by 1% agarose gel electrophoresis. All the DNA samples are stored at −20°C until sequencing. For 16S rRNA gene sequencing, the V3–V4 hypervariable regions of the 16S rRNA are amplified from microbial genomic DNA with primers 338F: ACTCCTACGGGAGGCAGCAG; 806R: GGACTACHVGGGTWTCTAAT using the hermocycler PCR system (GeneAmp 9700, ABI). PCR reactions are conducted according to the following program: 3 min of denaturation at 95°C, 28 cycles of 30 s at 95°C, 30 s for annealing at 55°C and 45 s for elongation at 72°C, and a final extension at 72°C for 10 min. The ITS2 hypervariable regions of fungal genes are amplified with primers ITS3F: GCATCGATGAAGAACGCAGC; ITS4R: TCCTCCGCTTATTGATATGC by thermocycler PCR system (GeneAmp 9700, ABI). The PCR reactions are conducted according to the following program: 3 min of denaturation at 95°C, 35 cycles of 30 s at 95°C, 30 s for annealing at 55°C and 45 s for elongation at 72°C, and a final extension at 72°C for 10 min. Purified amplicons of 16S rRNA or ITS2 sequences are pooled in equimolar and paired‐end sequenced (2 × 300) on an Illumina MiSeq platform (Illumina) following the standard protocols of Majorbio Bio‐Pharm Technology Co., Ltd.

For metagenome, we extract fecal DNA based on the OMEGA Mag‐Bind Soil DNA Kit (M5635‐02) (Omega Bio‐Tek) following the manufacturer's instructions. The concentration and purity of extracted DNA are assessed using a Qubit 4 Fluorometer with WiFi: Q33238 (Qubit Assay Tubes: Q32856; Qubit 1X dsDNA HS Assay Kit: Q33231) (Invitrogen) and agarose gel electrophoresis, respectively. The extracted DNA is kept at −20°C for further assessment. We process the extracted microbial DNA to construct metagenome shotgun sequencing libraries with insert sizes of 400 bp according to the Illumina TruSeq Nano DNA LT Library Preparation Kit. Each library is sequenced by the Illumina NovaSeq platform (Illumina) with the PE150 strategy at Personal Biotechnology Co., Ltd. with at least 10 GB of raw data per sample.

## STATISTICAL ANALYSIS PLAN

Descriptive data will be analyzed and presented using appropriate statistical methods. The CGM readings will be processed to generate different glycemic metrics, such as time in/above/blow target range, glucose area under the curve, and coefficient of variation. We will estimate the associations of CGM‐derived glycemic features with dietary intake, physical activity, and birth outcomes using regression analysis.

For genotyping data, variants will be mapped to the 1000 Genomes Phase 3 v5 by SHAP EIT [33, 34]. Genome‐wide genotype imputation will be conducted using the 1000 Genomes Phase 3 v5 reference panel by Minimac3 [35, 36]. We will use the genomic‐relatedness‐based restricted maximum‐likelihood method in a software tool called genome‐wide complex trait analysis to estimate the proportion of variance shown by all SNPs [37]. We will further perform a colocalization analysis of *cis*‐protein quantitative trait loci (pQTL) based on the genomics and proteomics data.

For microbiome analysis, we will compare the differences in *α*‐diversity indices, such as the Shannon index (a quantitative measure of community diversity), observed features (a qualitative measure of community richness), and the Gini–Simpson index (or the Simpson index, a measure of community evenness), between groups defined by different study aims using the Kruskal–Wallis test or regression analysis. Microbiome multivariable association with linear models [38] or multivariate regression will be used for the selection of taxonomic features.

Nonlinear dimensionality reduction such as uniform manifold approximation and projection or principal component analysis will be performed to summarize variations in each single omics data set. We will conduct further integrated analysis of different omics data sets using correlation analysis, regression analysis, machine learning (e.g., Light Gradient Boosting Machine), and pathway enrichment analysis.

## BASIC CHARACTERISTICS OF THE PARTICIPANTS AT RECRUITMENT

We have included 1715 pregnant women with GDM in WeBirth up to October 2022. Among these, for 1621 women, complete data of the basic characteristics were obtained at recruitment (Table 2). Participants' ages range from 21 to 44 years (mean = 31.2, SD = 3.7), with 15.7% (254/1621) being older than 35 years of age. The average pre‐pregnancy body mass index and gestational age at recruitment are 22.2 ± 3.6 kg/m^2^ and 26.0 ± 1.9 weeks, respectively. More than half of the pregnant women (67.7%) are primipara. Most of the participants (95.7%) never smoke. Only 2.7% of the participants are current alcohol drinkers, defined as those consuming a drink containing alcohol at least once a month on average in the past year. The participants have an average daily dietary intake of 1775 kcal (SD: 726 kcal) based on the FFQ.

**Table 2:** Basic characteristics of the participants at recruitment (up to October 2022).

	Total	Age <35 years	Age ≥35 years
Number of participants	1621	1367	254
Age, years	31.2 (3.7)	30.1 (2.7)	37.4 (1.9)
Pre‐pregnancy BMI, kg/m^2^	22.2 (3.6)	22.0 (3.6)	23.0 (3.6)
Gestational age at baseline, week	26.0 (1.9)	26.0 (1.9)	25.9 (1.9)
Parity			
0	1098 (67.7%)	1009 (73.8%)	89 (35.0%)
1	491 (30.3%)	341 (24.9%)	150 (59.1%)
2	30 (1.9%)	17 (1.2%)	13 (5.1%)
3	2 (0.1%)	0 (0.0%)	2 (0.8%)
Education			
≤High school or vocational school	183 (11.3%)	138 (10.1%)	45 (17.7%)
University or professional school	1208 (74.5%)	1030 (75.3%)	178 (70.1%)
>University	230 (14.2%)	199 (14.6%)	31 (12.2%)
Household income, ¥/year			
<100,000	355 (21.9%)	304 (22.2%)	51 (20.1%)
100,000–200,000	574 (35.4%)	485 (35.5%)	89 (35.0%)
>200,000	588 (36.3%)	488 (35.7%)	100 (39.4%)
Unclear^a^	104 (6.4%)	90 (6.6%)	14 (5.5%)
Smoking			
Never smoker	1552 (95.7%)	1310 (95.8%)	242 (95.3%)
Exsmoker	68 (4.2%)	56 (4.1%)	12 (4.7%)
Current smoker	1 (0.1%)	1 (0.1%)	0 (0.0%)
Current drinking	44 (2.7%)	35 (2.6%)	9 (3.5%)
Energy intake, kcal/day	1775 (726)	1776 (720)	1769 (759)
Physical activity, MET‐h/wk	19.4 (8.5)	19.2 (8.3)	20.3 (9.0)

*Note*: Data are mean (SD) or *n* (%).

Abbreviations: BMI, body mass index; MET, metabolic equivalent.

a Participants who refused to provide information on their household income.

## STRENGTHS AND LIMITATIONS

WeBirth is a prospective cohort study of ~2000 women participants with GDM and their offspring. Participants receive standardized testing meals and undergo objective measurements of physical activity and continuous blood glucose for 14 days, keeping timely dietary records during this period. We have applied strict quality control standards: quality control is maintained throughout the study to ensure high quality of data collection. As mentioned before, we set warnings for missing data or extremely outrageous value for each variable in the questionnaires. Biological samples are collected, aliquoted, and assessed following standardized protocols. Information on dietary habits and physical activity is collected using different methods (FFQ, 24‐HR, and daily dietary records; the PPAQ‐C and wearable accelerometer for monitoring physical activity), which can be used for mutual verification. Besides, the measurement of FPG during the 1‐day event and wearing of the CGM device on both two arms simultaneously can enable testing of the stability and quality of CGM readings. The unique study design enables us to explore the personalized glycemic response to nutrition and physical activity. Simultaneously, deep phenotyping depicts the atlas of personal characteristics at different scales, including physical measurements, lifestyle factors, psychosocial characteristics, medical records, clinical biomarkers, and multiomics molecular mapping.

The present study has several limitations. First, our cohort includes Chinese participants, and, therefore, the findings may not completely be generalizable to other ethnicities. Second, our study only includes participants with GDM, with the lack of a normal control group at this stage. Finally, owing to the observational nature of the cohort study, we could not establish causality based on the current cohort data.

In conclusion, WeBirth provides a new framework for a prospective birth cohort study with sophisticated integration of precision nutrition, wearable devices, and collection of multi‐omics data among patients with GDM. Data from WeBirth will represent a unique source, and will substantially contribute to our knowledge of precision nutrition for GDM management, risk factors for adverse birth outcomes, and factors influencing the long‐term health of pregnant women with GDM and their offspring.

## AUTHOR CONTRIBUTIONS

Ju‐Sheng Zheng was involved in the conceptualization, investigation, resource procurement, writing of the manuscript—review and editing, supervision, and funding acquisition of the study. Wen‐Sheng Hu was involved in the conceptualization, investigation, resource procurement, writing of the manuscript—review and editing, and funding acquisition of the study. Xinxiu Liang was involved in the formal analysis, investigation, resource procurement, data curation, writing of the manuscript—original draft, writing of the manuscript—review and editing, visualization, and project administration of the study. Zelei Miao was involved in the investigation, resource procurement, writing of the manuscript—review and editing, and project administration of the study. Sha Lu was involved in the resource procurement of the study and writing of the manuscript—review and editing. Meng Ye was involved in the formal analysis, investigation, and data curation of the study and writing of the manuscript—review and editing. Jiali Wang was involved in the investigation and resource procurement of the study and writing of the manuscript—review and editing. Hui Zhao was involved in the investigation of the study and writing of the manuscript—review and editing. Congmei Xiao was involved in the investigation of the study and writing of the manuscript—review and editing. Menglei Shuai was involved in the investigation, writing of the manuscript—review and editing, and visualization of the study. Wanglong Gou was involved in the investigation and resource procurement of the study, and writing of the manuscript—review and editing. Yuhui Liang was involved in the investigation and data curation of the study and writing of the manuscript—review and editing. Fengzhe Xu was involved in the investigation of the study and writing of the manuscript—review and editing. Mei‐Qi Shi was involved in resource procurement of the study and writing of the manuscript—review and editing. Ying‐Ying Wu was involved in resource procurement of the study and writing of the manuscript—review and editing. Xu‐Hong Wang was involved in resource procurement of the study and writing of the manuscript—review and editing. Feng‐Cheng Cai was involved in resource procurement of the study and writing of the manuscript—review and editing. Meng‐Yan Xu was involved in resource procurement of the study and writing of the manuscript—review and editing. Yuanqing Fu was involved in the investigation, resource procurement, writing of the manuscript—review and editing, and project administration of the study.

## CONFLICT OF INTEREST STATEMENT

The authors declared no conflict of interest.

## ETHICS STATEMENT

WeBirth was approved by the Ethics Committee of Westlake University (20190701ZJS0007). Written informed consent was obtained from each participant.

## Supporting information

Supporting information.

## Data Availability

Most of the data are not ready at this stage. Please contact the corresponding author (Ju‐Sheng Zheng) for the rawdata request. Supplementary materials (figures, tables, scripts, graphical abstract, slides, videos, Chinese translated version, and update materials) may be found in the online DOI or iMeta Science http://www.imeta.science/.
